# Nano-micelle Curcumin; A Hazardous and/or Boosting Agent? Relation with Oocyte *In-vitro* Maturation and Pre-implantation Embryo Development in Rats

**DOI:** 10.22037/ijpr.2019.14799.12671

**Published:** 2020

**Authors:** Moona Roshanfekr Rad, Vahid Nejati, Mazdak Razi, Gholamreza Najafi

**Affiliations:** a *Department of Biology, Faculty of Science, Urmia University, Urmia, Iran. *; b *Department of Basic Sciences, Faculty of Veterinary Medicine, Urmia University, Iran. *; c *Department of Anatomy and Embryology, Faculty of Veterinary Medicine, Urmia University, Iran.*

**Keywords:** Nano-micelle curcumin, Oocyte, Zygote, Blastocyst, Hatched embryo

## Abstract

The present study was done to uncover the possible beneficial and/or detrimental effect(s) of nano-micelle curcumin (NMC) on oocyte *in-vitro* maturation and pre-implantation embryo development. Forty-eight mature female Wistar rats were assigned to control, 7.5, 15, and 30 mg/kg^-1^ NMC-receiving (orally, for 48 days) groups. To assess the cumulus-oocyte complexes (COCs), the ovaries were stimulated by administrating (i.p.) a 25 IU of the pregnant mare’s serum gonadotropin (PMSG) hormone. Following 48-h, 15 IU of hCG was injected (i.p.), and the COCs were taken after 16-18-h. To analyze the pre-implantation embryo development ratio, the sperms were collected from clinically healthy male Wistar rats, and 3.0-3.6 × 106 per mL was added into the fertilization drop. The animals in 7.5 mg/kg^-1^ NMC-receiving group exhibited a higher oocyte number *versus* control and other NMC-receiving groups. The NMC, in a dose-dependent manner, decreased the Zygote, 2-cell, blastocyst percentages, as well as hatched embryos, compared to the control group (*P < *0.05). The 15 and 30 mg/kg^-1^ NMC-receiving groups represented a remarkable enhancement in type I arrest. Meanwhile, a significant (*P < *0.05) reduction was revealed in type III embryo arrest in the same groups. The NMC, at 7.5 mg/kg^-1^ potentially enhances the oocyte number, while it fairly reduces the pre-implantation embryo development, even when it is administrated in dose levels of 7.5 mg/kg^-1^ and/or higher. Although more studies are needed, the NMC could be considered as a suppressor of fertility potential, when consumed chronically even in low doses.

## Introduction

Curcumin, a hydrophobic polyphenolic compound with [1, 7-bis (4-hydroxy-3-methoxyphenyl)-1, 6heptadiene-3, 5-dione] formulation is commonly derived from the rhizome of the herb *Curcuma Longa*. The curcumin due to its anti-cancer, anti-inflammation, antioxidant, and anti-infectious properties, is widely used in various biological and medical fields (1-4). In line, the curcumin potentially reduces the lipid peroxides and augments the activity of antioxidant enzymes such as superoxide dismutase (SOD), catalase, glutathione peroxidase, and glutathione reductase, and via this mechanism, the curcumin exerts the reactive oxygen species (ROS) and reactive nitrogen species scavenging properties (5, 6). In another study, Yan-Der Hsuuw and co-workers (2005) have reported that the curcumin inhibits methylglyoxal-induced apoptosis in mouse embryonic stem cells and blastocysts (ESC-B5 cells), as well as the blastocyst, by inhibiting ROS-related derangements (7). 

In contrast to these findings, lastly the contrary effects of antioxidant agents, chemicals and/or drugs have been reported, as well. Accordingly, Shinde and co-workers (2015) have shown that the curcumin, at dose levels of 10 and 20 mg/kg, alters the menstrual cycle’s physiologic time, and significantly reduces the secondary follicles distribution in rats (8). In the other trials, it has been demonstrated that the curcumin-PEG significantly down-regulates the follicular growth and exerts estrogen-like transcriptional activity (9, 10). Chen *et al.* (2010) showed that the curcumin (at a dose level of 24 mM) adversely affects the mouse blastocyst generation via inducing mitochondria-dependent apoptosis (11). In the line with this experimental trial, Chia-Chi Chen and Wen-Hsiung Chan have reported that consumption of drinking water containing 40 μM of curcumin potentially diminishes the oocyte maturation, as well as a pre-implantation embryo, development (12). Recently, it has been shown that the curcumin exerts various promoting effects on the female reproductive system, such as reducing the cancer risk factors, while it potentially exerts pro-apoptotic effects, as well (13). 

In spite of having such an extensive range of therapeutic and antioxidant properties, the major difficulty associated with the utility of curcumin is its low bioavailability upon administration (3, 5). For overcoming this problem, the natural curcumin has been converted to a nano-micelle form, which remarkably enhances its bioavailability, absorption, and clinical features (2). In the line with this issue and using rats as animal model, we showed that the curcumin in the form of nano-micelle remarkably up-regulates the pro-apoptotic genes p53 and caspase-3, and down-regulate the anti-apoptotic gene Bcl-2 expressions in germinal epithelium of the male rats, and significantly lowers the sperm quality, and ultimately inhibits the pre-implantation embryo development (14, 15). 

Based on bolded positive properties of antioxidants and oversimplifying of their hazardous effects, the public confidence to higher consumption of curcumin and its nano-changed types, without concerning their possible hazardous impact, has been increased. Thus, identifying the side effects of potent antioxidant chemicals and supplements have become a major challenge in the field of reproductive medicine. Therefore, the current study was designed to investigate the dose-dependent effect of nano-micelle curcumin (NMC) on oocyte *in-vitro* maturation (IVM), fertilization ratio, and pre-implantation embryo development using an animal model study. 

## Experimental

*Animals*

Thus, to follow-up the current study, 48 mature (180 ± 15 g, 6-8 weeks old) virgin female Wistar rats were randomly divided into control and experimental groups. The animals were kept in a standard condition (12-h light/12-h dark cycle, 21 ± 01 °C). All animals received the standard rat diet and water ad libitum. Following 48 days, the animals were euthanized by ketamine and xylazine (Alfasan, Woerden, The Netherland) overdose administration. All analyses and investigations were conducted based on the guideline for animals care and research of Urmia University (Ethical No: 2/PD/85). The mature, virgin and clinically healthy rats were included in the study. The animals with anatomically visible abnormalities, as well as those immature, pregnant and weak animals, were excluded in the current study. E = Total number of animals - Total number of groups was considered as a sample size formula. 

*Experimental design*

Our previous experimental trial showed that administration of 7.5 mg/kg^-1^, 15 mg/kg^-1^ and 30 mg/kg^-1^ NMC in the male Wistar rats, negatively and dose-dependently affected the embryo development process. To see the effect of NMC on female reproductive potential, here in the current study, the animals in experimental groups were subdivided into 4 groups and received 7.5 mg/kg^-1^ (low dose), 15 mg/kg^-1^ medium dose) and 30 mg/kg^-1^ (high dose) of NMC (No: 12 rats in each group of total 4 groups, including control). The animals in control group received the same volume of solvent (saline normal), which was used for NMC. The chemicals were administrated orally. 

*Germinal vesicle (GV) oocytes dissection for IVM and in-vitro fertilization*

Following 48 days, in order to take GV oocytes, 25 IU (i.p.) of the pregnant mare’s serum gonadotropin (PMSG, ASKA Pharmaceutical, Tokyo, Japan) hormone was administrated. Following 48-h, the hCG (15 IU) was injected (i.p.) and thereafter followed to collect the GV oocytes after 16-18-h (16). The animals were euthanized to collect the GV oocytes, the ovaries were dissected out and placed in Petri dishes containing TCM199 medium (Gibco, Invitrogen, USA, Cat NO: 11150059) supplemented with 5% (v/v) heat-inactivated fetal bovine serum (Merck, Germany, Cat NO: F2442). Cumulus-oocyte complexes (COCs) were released by follicular puncturing with the 30 G needles under high magnification provided by a stereomicroscope (Olympus, Japon). Only immature oocytes containing compact cumulus cells were considered. Next, by using 0.1% hyaluronidase in PB1 medium, the oocytes were freed from cumulus cells (17). 

*Preparing IVM culture media and activation of oocytes after IVM*

A day pre-conception, the required culturing mediums were prepared and incubated in 5% CO_2_ and 37 °C. To prepare the culture media for IVM, TCM199, which was remixed with FBS 10% (Merck, Germany, Cat NO: F2442), 100 mIU of FSH and 40 mIU of LH (Merck, Germany, Cath NO: L6420-10UG) in 1 mL of medium. After mixing, the 50 µL drops were transferred into a dish and covered with mineral oil (18). After culturing *in-vitro*, artificial parthenogenetic activation was carried out with oocytes that reached MII. The MII oocytes were physically denuded with a glass capillary pipette. The oocytes were then cultured in the previously prepared medium. After culturing, the oocytes that contained a distinct pronucleus were counted and compared between the groups.

*In-vitro-fertilization (IVF) process and comparing data*

A day pre-fertilization, the mR1ECM culture medium was incubated at 12-h in 5% Co_2_ in 37 °C. In order to perform IVF, 10-20 oocytes were transferred into each individual drop (500 µL from culture media) and then 10 µL of the sperm medium (containing 3.0-3.6 × 10^6^ sperms) was allocated. The percentages of the zygote and pre-implantation 2-cell, blastocyst, and hatched embryos were estimated at 24-h, 4, and 5 days after IVF, respectively (19).

*Evaluation of embryo development*

The *in-vitro* development was evaluated and the intact and fragmented and/or lysed embryos that did not continue developing were recorded as “arrested embryos”. The following type of embryo arrest was considered: Type I: fully lysed, necrotic and/or fragmented embryos. Type II: embryos with partially lysed/ fragmented blastomeres. Type III: embryos with lysed and/or fragmented blastomeres, and embryos with cytoplasmic vesicles. The percentage of each type was assessed in each experimental group and compared between the groups. By quantification of non-compacted, compacted morulae, early blastocysts (with initial blastocele), expanded blastocysts, hatching blastocysts (zona-escaping blastocyst), and hatched blastocysts (extruded or zona-free embryos) the embryo differentiation was examined.

*Study limits and strengths*

There are some limitations in the current study. Although analyzing the *in-vitro* fertilization potential and/or pre-implantation embryo development helps us to conclude about the possible effect of NMC on the fertilization status, the effect of NMC on follicular growth, and/or atresia will illustrate the effect of NMC on ovarian physiology and folliculogenesis. Moreover, the effect of NMC on hypophysis-ovary axis-sourced gonadotropins, by assessing the serum levels of FSH and LH, as well as ovarian-sourced hormones, such as estrogen and progesterone should be considered in other studies. However, estimating the GVs, fertilization potential, and pre-implantation embryo development were investigated in the current trial, which first, can be helpful for uncovering the dose effect of NMC on mentioned parameters, and second, can be helpful when the results are possibly considered for human cases. 

*Statistical analyses*

All data were analyzed with One-way ANOVA; a Duncan multiple-comparison test was used to locate differences. For this purpose, the SPSS software (version 11.5) was used. The data are expressed as the mean ± SD, and the *P* < 0.05 was considered to be statistically significant.

## Results

*Oocyte number and GV results*

**Figure 1:**
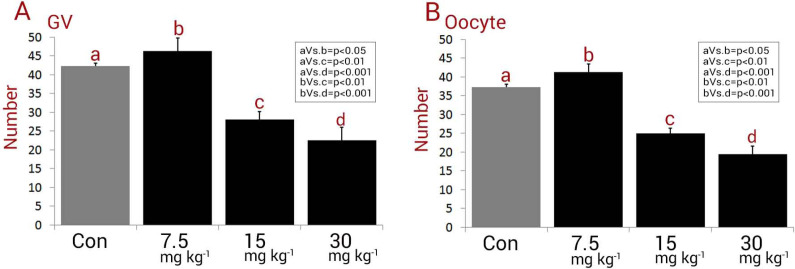
Mean numbers of (A) germinal vesicles (GV) and (B) oocytes in different groups, all data are presented in mean ± SD. Different letters are representing significant differences between groups, Con: Control. Note decreased GV and oocyte number in 15 mg kg^-1^ and 30 mg kg^-1^-receiving groups versus control animals

Observations revealed a significant (*P < *0.05) reduction in GV and oocyte numbers in NMC-receiving animals. Accordingly, the rats in medium and high dose NMC-receiving groups exhibited a remarkable reduction in GV and oocyte numbers *versus* control and low dose NMC-receiving groups. The animals in the low dose NMC-receiving group represented significantly (*P < *0.05) higher GV and oocyte numbers when compared to those in the control group. Therefore, the total GV and oocyte numbers in low dose NMC-receiving group were significantly higher *versus* the control group (Figures 1A and 1B). 

*Oocyte fertilization and pre-implantation embryo development*

**Figure 2:**
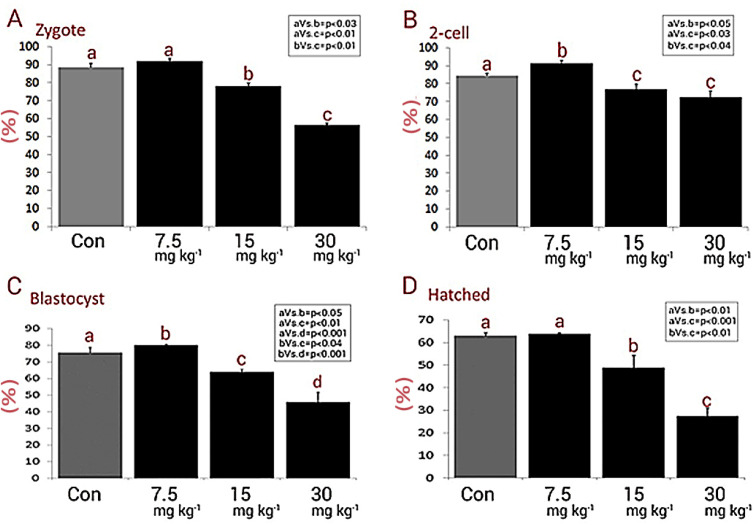
Mean percentages of (A) zygotes, (B) 2-cell, (C) blastocysts and (D) hatched embryos in different groups, all data are presented in mean ± SD. Different letters are representing significant differences between groups, Con: Control. The NMC significantly diminished pre-implantation embryo development (especially at higher doses) compared to control group

**Figure 3:**
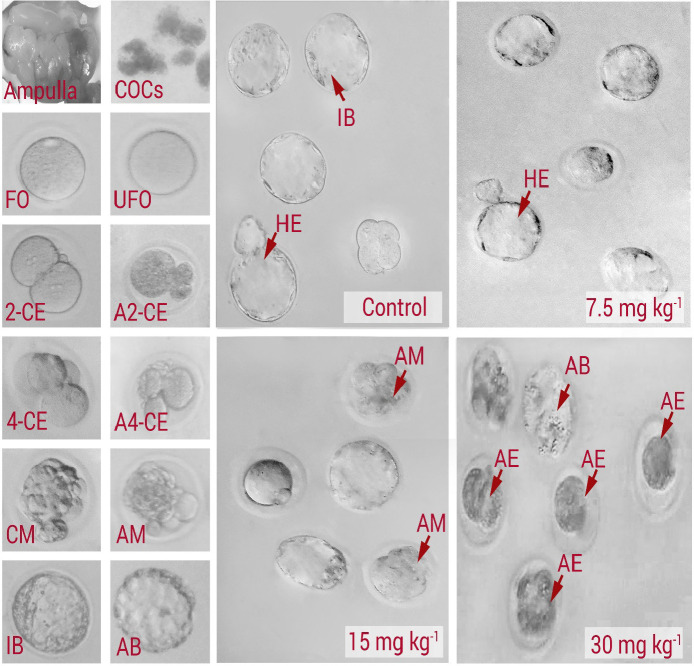
Phase-contrast microscopic view of pre-implantation embryos: Control, 7.5 mg/kg^-1^ NMC-receiving group (low dose), 15 mg/kg^-1^ NMC-receiving group (medium dose), and 30 mg/kg^-1^ NMC-receiving group (high dose). COCs: cumulus-oocyte complexes; FO: Fertilized oocyte; UFO: unfertilized oocyte; 2-CE: 2-cell embryo; A2-CE: arrested 2-cell embryo; 4-CE: 4-cell embryo; A4-CE: arrested 4-cell embryo; CM: compact morula; AM: arrested morula; IB: intact blastocyst; AB: arrested blastocyst; HE: hatched embryo. See normal embryo development in control group with high rate of blastocyst, which is changed with arrested embryoes at different stages of development in NMC-receiving groups. Accordingly, the drop containing embryoes in 30 mg kg^-1^ group represents severe embryonic arrest at early stages of development

In order to assess the effect of NMC on fertilization potential, the percentages of zygotes in different groups were estimated. The animals in the medium and high dose NMC-receiving groups exhibited a remarkable (*P < *0.05) reduction in percentages of zygotes *versus* control and low dose NMC-receiving animals (Figures 2A and 2B). No significant changes were noted between the low dose NMC-receiving and control groups (*P *> 0.05). More analyses were conducted to assess the effect of NMC on pre-implantation embryo development. In line, the animals in the low dose NMC-receiving group exhibited a significant (*P < *0.05) up-regulation in percentages of 2-cell embryos, as well as the blastocysts, *versus* the control group. However, this situation was reversed in medium and high dose NMC-receiving groups. Accordingly, the animals in medium and high dose NMC-receiving groups exhibited a remarkable (*P < *0.05) reduction in percentages of 2-cell embryos and blastocysts when compared to the control group (Figure 2C). Finally, the percentages of hatched embryos were compared with control group, and observations demonstrated no significant changes in the low dose NMC-receiving animals and revealed a remarkable reduction in medium and high dose NMC-receiving groups (Figure 2D). The photomicrographs of the embryos in different stages are represented in Figure 3.

*Embryonic arrest*

**Figure 4:**
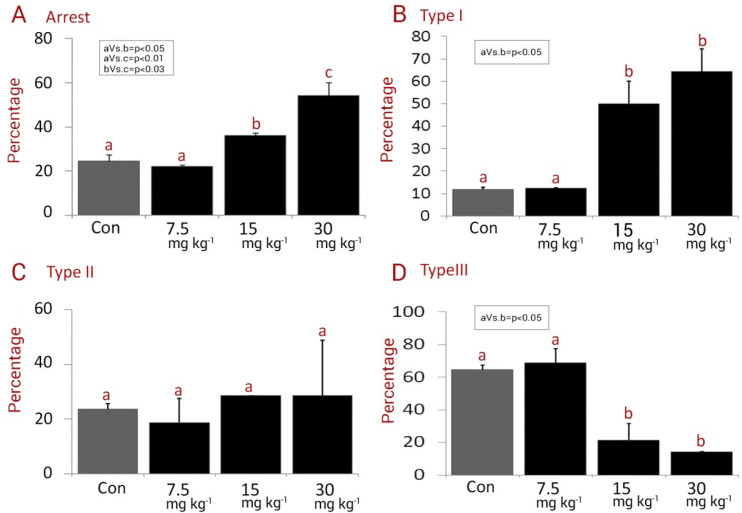
Mean percentages of (A) Arrest, (B) Arrest type I, (C) Arrest type II and (D) Arrest type III in different groups. all data are presented in mean ± SD. Different letters are representing significant differences between groups, Con: Control

The data for total and three types of arrests are represented in Figure 4. No significant differences in the total percentages of arrested embryos were revealed between the low dose NMC-receiving group and control groups (*P *> 0.05). Meanwhile, the animals in medium and high dose NMC-receiving groups exhibited remarkably (*P < *0.05) higher total embryonic arrest compared to control and low dose NMC- receiving groups (Figure 4A). In more details, no statistically remarkable changes were observed for three types of arrests between the control and low dose NMC-receiving animals (*P *> 0.05). In contrast, all three types of arrests were increased significantly (*P < *0.05) in the medium and high dose NMC-receiving groups (Figures 4B-4D)

## Discussion

Our results showed that the low dose NMC-receiving rats (7.5 mg kg ^-1^) exhibited higher GV and oocytes numbers *versus* control and other experimental groups. Moreover, the low dose NMC significantly enhanced the percentages of 2-cell embryos and blastocysts and exerted no significant effect on the percentages of arrested and hatched embryos when compared to the control group. In contrast, the NMC, at higher doses (15 and 30 mg kg^-1^), significantly diminished the pre-implantation embryo development, increased the embryonic arrest, and finally lowered the hatched embryos percentage *versus* control and low dose NMC-receiving animals. 

As preliminary findings, our data represented a remarkable enhancement in numbers of oocytes and zygote in the low dose NMC-receiving group, as well as a considerable reduction in the medium and high dose NMC-receiving groups. To uncover the dose-dependent effect of NMC on oocytes number, as well as GV numbers, one should note the cross-links between ovulation and ovarian antioxidant capacity during pre-ovulatory stages. The meiosis I resume in dominant follicles of the ovaries in each menstrual cycle. Indeed, the suppressed ovarian antioxidant status and enhanced free radical generation maintain the meiosis I ratio. However, the meiosis II, is majorly promoted by up-regulation of ovarian antioxidant status, which finally results in physiologic ovulation. Thus, the balance between oxidants and antioxidant potential resumes/maintains the folliculogenesis and ovulation (20). In line, it should be noted that the increment of aromatization and p450 expression/activity result in massive free radicals generation, which ends in ovulation following ovarian antioxidant system in-situ up-regulation (21). Our findings showed that the animals in the low dose NMC-receiving group exhibited enhanced oocyte number *versus* the control group, while the animals in medium and high dose NMC-receiving groups represented diminished numbers of oocytes *versus* control group. Considering the findings above we can come close to this fact that the NMC, at higher dose levels, adversely affects the dominant follicles (those follicles in meiosis I) growth and population, and consequently decreases the ovulation ratio. 

As a second theory, it should be considered that the estrogen plays an essential role in ovulation and any disruption in the synthesis of estrogen is able to potentially suppress the ovulation (20). Indeed, the estrogen increases in response to physiologic amounts of follicle stimulating hormone (FSH) and triggers the expression/synthesis of catalase (as an endogenous antioxidant enzyme). In turn, the catalase protects the dominant follicles grow up and significantly survives these follicles from apoptosis (22). Moreover, there is a positive correlation between estrogen concentration and the number of ovarian follicles. Accordingly, at the proliferative stage, the growing follicles produce increasing levels of estrogen. In line with this theory, several previous reports have shown that the curcumin at dose levels of higher than 10 mg kg b.w^-1^ exhibits antiestrogenic effects (8, 23). Thus, we can come close to this fact that the 15 and 30 mg kg^-1^ of NMC, as a potentially more active chemical *versus* natural curcumin, could fairly down-regulate the follicular grow up, and consequently suppressed the estrogen synthesis and resulted in a significant reduction in ovulation ratio. 

Indeed, the oocyte viability, maturation, and pre-implantation embryo development have been shown to mainly depend on glucose contents and ROS concentration in the microenvironment (24, 25). Chen and co-workers (2010) showed that incubation of blastocysts with 2, 4, 6 and 12 µm of curcumin significantly enhances reactive oxygen species (ROS) generation and triggers the mitochondria-dependent apoptosis (11). During physiologic embryogenesis, albeit after the blastocyst stage, the apoptosis removes the abnormal and redundant cells, leading to embryo development arrest because of DNA fragmentation and/or mutation (7, 25). On the other hand, the early apoptosis (mainly through mitochondria-dependent pathway) of pre-implantation embryos (mainly at stages of 2-cells and 8-cells) has been shown to positively correlate with oxidative stress (7, 26-28). Minding these issues, it could be suggested that the NMC (at dose levels of 15 and 30 mg kg^-1^) was able to potentially induce oxidative stress, and via this mechanism, it could result in embryo development arrest. However, it exerts a contradictory effect, when it is administrated at the dose level of 7.5 mg kg^-1^.

We tried to show the adverse effect of NMC on preblastocyst as well as hatched embryos. Our findings showed that the NMC, albeit at medium and high dose levels, significantly elevated the percentages of embryos with type I, II, and III arrests, and remarkably decreased the hatched embryos percentages. To understand the subject one should note that the late embryonic cells death, as one of the main reasons of late embryo arrest, largely depends on nuclear and cytoplasmic fragmentation and/or mitochondria-dependent apoptosis (29). On the other hand, several previous studies have shown that the curcumin initiates the mitochondria-dependent apoptosis via triggering the caspase-3 overexpression, down-regulating bcl-2 expression (30, 31). Considering the fact that curcumin is able to potentially induce mitochondria-dependent apoptosis and minding the NMC-induced embryo arrest, we can come close to this fact that the NMC (additional to oxidative stress) is able to result in problastocysts arrest by inducing apoptosis. 

## Conclusion

Our data demonstrated that the NMC (at higher dose levels) is able to potentially reduce the total available oocytes number and *in-vitro* maturation ratio. Moreover, chronic consumption of high doses of NMC suppresses the *in-vitro* pre-implantation embryo development. However, more studies are needed to show the mechanisms, in which the NMC is able to reduce the oocyte quality as well as embryo development. 
